# Comparison of Intraocular Cytokine Levels of Choroidal Neovascularization Secondary to Different Retinopathies

**DOI:** 10.3389/fmed.2021.783178

**Published:** 2021-12-21

**Authors:** Chenyi Liu, Shian Zhang, Xinyi Deng, Yijing Chen, Lijun Shen, Liang Hu, Jianbo Mao

**Affiliations:** ^1^School of Optometry and Ophthalmology, Wenzhou Medical University, Wenzhou, China; ^2^Eye Hospital of Wenzhou Medical University, Wenzhou, China

**Keywords:** choroidal neovascularization, age-related macular degeneration (AMD), polypoidal choroidal vasculopathy (PCV), high myopic, central serous chorioretinopathy (CSC), cytokine, vascular endothelial growth factor

## Abstract

**Purpose:** To investigate and compare the aqueous concentrations of vascular endothelial growth factor (VEGF) and other inflammatory cytokines in various choroidal neovascularization (CNV) diseases and types.

**Methods:** This observational study included 127 naive eyes with CNV and 43 control eyes with cataracts. Aqueous humor (AH) samples were obtained prior to intravitreal anti-VEGF injection or cataract surgery. Multiple inflammatory cytokines, including VEGF, interleukin (IL) 6, IL-8, IL-10, interferon-inducible protein 10 (IP-10), and monocyte chemotactic protein 1 (MCP-1) levels, were measured using a multiplex bead assay. The angiogenesis index was defined as the ratio of IP-10 to MCP-1. In addition, the relationship among AH cytokine levels, central macular thickness (CMT), and CNV size on optical coherence tomography angiography (OCTA) was evaluated.

**Results:** Except in the myopic CNV group (*P* = 0.452), the AH concentration of VEGF was significantly higher in all other CNV groups than in the control group (*P* < 0.05 for all comparisons). IL-8, IL-10, IP-10, and MCP-1 levels (*P* < 0.05 for all groups) were significantly higher in all CNV diseases except those with neovascular central serous chorioretinopathy. The angiogenesis index was significantly higher in all CNV diseases (*P* < 0.05 for all comparisons). The VEGF level may be associated with the size of the CNV on OCTA (*p* = 0.043).

**Conclusions:** The level of intraocular inflammatory cytokines varied among different CNV diseases and CNV types. Therefore, the angiogenesis index may be a more sensitive indicator of angiogenesis.

## Introduction

Choroidal neovascularization (CNV) is the formation of new blood vessels in the choroid. It often occurs in the macular area, causing macular hemorrhage and serous exudation under the retina, which can result in blindness. CNV is a characteristic finding in many fundus diseases, such as age-related macular degeneration (AMD), pathologic myopia, polypoidal choroidal vasculopathy (PCV), and central serous chorioretinopathy (CSC) (1, 2).

Although the pathogenesis of CNV remains poorly understood, it is believed to involve a variety of cell growth factors, and the angiogenesis is controlled by a dynamic equilibrium between proangiogenic and anti-angiogenic cytokines (2). Vascular endothelial growth factor (VEGF) is considered the most critical regulator in ocular angiogenesis (3). Currently, intravitreal anti-VEGF injections are recommended as the first-line treatment in patients with neovascular age-related macular degeneration (nAMD). However, anti-inflammatory therapy has also achieved curative efficacy in patients with neovascularization (4–6). It is still unclear whether the neovascularization in various CNV diseases results from the elevation in the VEGF concentration or the presence of the inflammatory response, and the application of anti-inflammatory treatment remains controversial (7–10).

CNVs are classified into Type 1 and Type 2 CNVs depending on the anatomic localization of the neovascularization. Further research is warranted to discover the aqueous concentration of cytokines in these two neovascularization types and investigate the potential mechanisms linked to their inflammatory response. In addition, CNV size was found to be associated with functional prognosis, suggesting that it could predict the response to anti-VEGF therapy (11). However, it is unknown whether the reason is related to VEGF or inflammatory cytokines.

Secondary CNV was reported in 5% of chronic CSC cases in which the concentration of VEGF did not increase (12, 13). Previous studies have not investigated the change in VEGF levels or the difference in cytokine profiling between neovascular CSC (nCSC) and other CNV diseases.

This study investigated and compared the aqueous concentrations of VEGF and other inflammatory cytokines in different CNV diseases and CNV types. Additionally, it preliminarily explored the association between VEGF levels and the size of CNV on optical coherence tomography angiography (OCTA) images.

## Methods

This observational study was conducted between May 2019 and July 2021 and included 127 eyes of patients treated for active CNV in nAMD, PCV, myopic choroidal neovascularization (mCNV), and nCSC. This study was approved by the Wenzhou Medical University Affiliated Eye Hospital Ethics Committee, and the procedures followed the tenets of the Declaration of Helsinki (IRB number #121-K-107-01). All patients provided written informed consent for inclusion in the study.

The inclusion criteria were (1) age over 18 years, (2) no previous history of intraocular surgery or intravitreal injections, (3) CNV in the active stage diagnosed by fluorescein angiography and indocyanine green angiography, and (4) a spherical equivalent of the eyes with mCNV ≤ −6.00 D and an axial length of >26 mm in patients with high myopia. The exclusion criteria were (1) any other retinopathy such as diabetic retinopathy, retinal vascular occlusion or retinal detachment (2) glaucoma and/or iris neovascularization; (3) uveitis; and (4) any previous treatment for CNV, including laser treatment within the past 6 months. The control group consisted of 43 eyes that underwent cataract surgery and had no other ocular or immune-mediated diseases.

All patients received a comprehensive ophthalmological examination before their anti-VEGF injection or cataract surgery, including slit lamp biomicroscopy and dilated fundus examination. The axial length was measured using the IOL-Master 700 (Carl Zeiss Meditec, Jena, Germany). The OCTA images were obtained using a spectral domain optical coherence tomography (SD-OCT) device (RTVue XR Avanti, Optovue, Inc., Fremont, CA, USA) with a split-spectrum amplitude-decorrelation angiography algorithm. The size of CNV was marked with the manual selection tools and calculated using the built-in software. The image quality for analysis was no lower than 7 out of 10. All measurements were performed by two independent and masked readers. Disagreements over readings were resolved by open adjudication between readers. The CNV size was classified as small (<0.5 mm^2^), medium (≥0.5 mm^2^ and <2.0 mm^2^), and large (≥2.0 mm^2^).

CNVs were categorized into two groups, Type 1 and Type 2, depending on their anatomical location. Type 1 CNV was characterized by any abnormal vasculature localized between Bruch's membrane and the retinal pigment epithelial on SD-OCT, and Type 2 CNV was characterized by any abnormal vasculature localized above the retinal pigment epithelium and beneath the photoreceptor outer segments on SD-OCT. Central macular thickness (CMT) was defined as the average retinal thickness within the 1 mm-diameter central field of the Early Treatment Diabetic Retinopathy Study.

Aqueous humor (AH) samples were collected before cataract surgery or intravitreal anti-VEGF injections. Approximately 0.05 mL of AH was withdrawn using a 30 gauge insulin syringe via limbal paracentesis. Each AH sample was immediately transferred into a sterile plastic tube and kept at −84°C until the assay. The levels of VEGF, interleukin (IL) 6, IL-8, IL-10, interferon-inducible protein 10 (IP-10), and monocyte chemotactic protein 1 (MCP-1) were measured in undiluted AH samples using a Luminex 200 (BIO-RAD, Hercules, CA, USA). Each cytokine concentration (pg/mL) was calculated using the standard curve provided by the kit. All steps were performed at room temperature and in dark illumination to protect the samples from light-induced degradation. The angiogenesis index was defined as the ratio of IP-10 to MCP-1.

All statistical analyses were performed using SPSS for Windows (version 26.0, SPSS Inc., Chicago, IL, USA). Continuous variables are expressed as means ± standard deviation. The normality of the cytokine data distribution was assessed using the Shapiro–Wilk test. For data that were not normally distributed, comparisons between groups were performed by non-parametric analysis of variance with either the Mann–Whitney U test or the Kruskal–Wallis test. For the Kruskal–Wallis test, significant values were adjusted using the Bonferroni correction for multiple tests of continuous variables. *P* < 0.05 was considered statistically significant.

## Results

In this observational study, 127 patients with CNV (nAMD = 47, PCV = 37, mCNV = 30, and nCSC = 13) and 43 control patients with cataracts were enrolled. The demographic features are summarized in Table 1.

**Table 1:** Differences in cytokine concentrations (pg/mL) between CNV groups and controls.

	**Control**	**nAMD**	**PCV**	**mCNV**	**nCSC**
	**(*n* = 43)**	**(*n* = 47)**	**(*n* = 37)**	**(*n* = 30)**	**(*n* = 13)**
Age	70.9 ± 7.3	71.3 ± 10.8	65.6 ± 11.6	57.6 ± 16.0	51.3 ± 9.2
Sex (M/F)	14/29	12/35	13/24	9/21	7/6
VEGF	29.78 ± 11.76	41.65 ± 19.06	40.31 ± 21.29	32.02 ± 14.49	36.99 ± 10.86
		0.004	0.007	0.452	0.044
IL-6	6.99 ± 7.89	6.50 ± 12.89	6.65 ± 10.42	12.23 ± 16.20	2.13 ± 0.91
		0.246	0.44	0.052	0.021
IL-8	9.37 ± 5.90	14.48 ± 19.69	34.95 ± 58.96	19.10 ± 13.08	4.87 ± 1.40
		0.262	0.018	<0.001	0.007
IL-10	0.68 ± 0.23	1.02 ± 0.46	1.37 ± 1.60	0.93 ± 0.18	0.69 ± 0.09
		<0.001	<0.001	<0.001	0.299
IP-10	178.87 ± 105.13	380.62 ± 266.52	709.68 ± 659.11	580.84 ± 513.65	225.07 ± 128.10
		<0.001	<0.001	<0.001	0.165
MCP-1	452.93 ± 193.11	494.82 ± 403.46	701.84 ± 549.77	752.72 ± 478.40	319.68 ± 71.62
		0.881	0.013	<0.001	0
Angiogenesis Index	0.42 ± 0.27	0.82 ± 0.47	1.08 ± 0.56	0.76 ± 0.39	0.70 ± 0.37
		<0.001	<0.001	<0.001	0.003

*nAMD, neovascular age-related macular degeneration; PCV, polypoidal choroidal vasculopathy; mCNV, myopic choroidal neovascularization; nCSC, neovascular central serous chorioretinopathy; VEGF, vascular endothelial growth factor; IL-6, interleukin 6; IL-8, interleukin 8; IL-10, interleukin 10; IP-10, interferon-inducible protein 10; MCP-1, monocyte chemotactic protein 1. P-values calculated by Spearman rank correlation analysis. P-values calculated by Mann–Whitney U-test*.

### VEGF Levels Were Higher in Most CNV Diseases

The AH concentrations of cytokines in all groups are presented in Table 1. The VEGF levels in the CNV groups (except the mCNV group, *P* = 0.452) were significantly higher than that in the control group (*P* < 0.05 for all comparisons, Table 1). In the AMD group, the levels of IL-10 and IP-10 were higher than those in the control group (*P* < 0.001 for both comparisons). In PCV and mCNV groups, the levels of IL-8, IL-10, IP-10, and MCP-1 were significantly higher than that in the control group (*P* < 0.05 for all comparisons). In addition, the angiogenesis indices in all CNV groups were significantly higher than that in the control group (*P* < 0.05 for all comparisons). No correlation was observed between cytokine levels and CMT (*P* > 0.05 for all comparisons, Table 2).

**Table 2:** Correlation between cytokines concentrations (pg/mL) and CMT at baseline.

	**VEGF**	**IL-6**	**IL-8**	**IL-10**	**IP-10**	**MCP-1**
nAMD	−0.196	−0.116	0.078	0.152	0.143	0.074
	0.238	0.488	0.64	0.364	0.39	0.658
PCV	0.161	−0.019	−0.129	0.007	0.007	0.04
	0.357	0.915	0.462	0.97	0.967	0.821
mCNV	0.065	0.349	−0.076	0.01	−0.206	−0.087
	0.757	0.087	0.717	0.963	0.324	0.68
nCSC	0.133	−0.35	−0.427	0.018	−0.25	−0.65
	0.732	0.356	0.252	0.963	0.516	0.058

*CMT, central macular thickness; nAMD, neovascular age-related macular degeneration; PCV, polypoidal choroidal vasculopathy; mCNV, myopic choroidal neovascularization; nCSC, neovascular central serous chorioretinopathy; VEGF, vascular endothelial growth factor; IL-6, interleukin 6; IL-8, interleukin 8; IL-10, interleukin 10; IP-10, interferon-inducible protein 10; MCP-1, monocyte chemotactic protein 1. P-values calculated by Spearman rank correlation analysis*.

### Intraocular Inflammatory Cytokine Levels in PCV Were Quite Different From Those in AMD

In the AMD Type 2 group, the levels of VEGF and IL-10 were significantly higher than those in the AMD Type 1 group (*P* < 0.05 for both comparisons). In the PCV group, the levels of IL-8, IL-10, and MCP-1 were higher than those in the AMD Type 1 group, but the levels of VEGF were lower than those in the AMD Type 2 group (*P* < 0.05 for all comparisons). Moreover, the level of IP-10 in the PCV group was significantly higher than that in the AMD Type 1 and AMD Type 2 groups (*P* < 0.05 for both comparisons, Table 3).

**Table 3:** Differences in cytokine concentrations (pg/mL) among PCV groups and nAMD Type1/Type 2 groups.

	**nAMD Type 1 (*n* = 27)**	**nAMD Type 2 (*n* = 20)**	**PCV**	**Type 1 vs. Type 2**	**Type 1 vs. PCV**	**Type 2 vs. PCV**
			**(*n* = 37)**			
VEGF	36.50 ± 18.12	48.60 ± 18.49	40.31 ± 21.29	0.024	0.348	0.037
IL-6	7.63 ± 16.43	4.98 ± 5.39	6.65 ± 10.42	0.966	0.833	0.841
IL-8	10.55 ± 7.76	19.79 ± 28.36	34.95 ± 58.96	0.156	0.039	0.488
IL-10	0.88 ± 0.30	1.20 ± 0.56	1.37 ± 1.60	0.019	0.028	0.718
IP-10	346.99 ± 245.80	426.02 ± 292.43	709.68 ± 659.11	0.159	<0.001	0.026
MCP-1	506.60 ± 483.30	478.90 ± 272.01	701.84 ± 549.77	0.445	0.007	0.091
Angiogenesis Index	0.75 ± 0.41	0.92 ± 0.54	1.08 ± 0.56	0.2	0.005	0.192

*nAMD, neovascular age-related macular degeneration; PCV, polypoidal choroidal vasculopathy; VEGF, vascular endothelial growth factor; IL-6, interleukin 6; IL-8, interleukin 8; IL-10, interleukin 10; IP-10, interferon-inducible protein 10; MCP-1, monocyte chemotactic protein 1. P-values calculated by Mann–Whitney U-test*.

### The Same Types of CNV Secondary to Different Causes Were Not Completely Similar in Cytokine Levels

Type 1 CNV was observed in 40 eyes (27 eyes with AMD and 13 eyes with CSC). The levels of IL-8, IL-10, and MCP-1 in eyes with AMD Type 1 were higher than those in eyes with CSC (*P* < 0.05 for all comparisons, Table 4). Type 2 CNV was observed in 47 eyes (20 eyes with AMD and 27 eyes with mCNV). The levels of IL-6 and MCP-1 in eyes with AMD Type 2 were lower than those in eyes with mCNV, but the level of VEGF was higher (*P* < 0.05 for all comparisons, Table 3).

**Table 4:** Differences in cytokine concentrations (pg/mL) of CNV diseases in the same Type 1 or Type 2 CNV.

	**Type 1**			**Type 2**		
	**nAMD (*N* = 27)**	**nCSC (*N* = 13)**	***P*** **-value**	**nAMD (*N* = 20)**	**mCNV (*N* = 27)**	***P*** **-value**
VEGF	36.50 ± 18.12	36.99 ± 10.86	0.475^b^	48.60 ± 18.49	31.65 ± 12.85	0.001^a^
IL-6	7.63 ± 16.43	2.13 ± 0.91	0.151^b^	4.98 ± 5.39	12.63 ± 16.87	0.009^b^
IL-8	10.55 ± 7.76	4.87 ± 1.40	0.006^b^	19.79 ± 28.36	19.35 ± 13.79	0.102^b^
IL-10	0.88 ± 0.30	0.69 ± 0.09	0.005^a^	1.20 ± 0.56	0.93 ± 0.19	0.031^b^
IP-10	346.99 ± 245.80	319.68 ± 71.622	0.120^b^	426.02 ± 292.43	589.25 ± 539.16	0.451^b^
MCP-1	506.60 ± 483.30	319.68 ± 71.62	0.019^b^	478.90 ± 272.01	763.92 ± 500.56	0.001^b^

*nAMD, neovascular age-related macular degeneration; mCNV, myopic choroidal neovascularization; nCSC, neovascular central serous chorioretinopathy; VEGF, vascular endothelial growth factor; IL-6, interleukin 6; IL-8, interleukin 8; IL-10, interleukin 10; IP-10, interferon-inducible protein 10; MCP-1, monocyte chemotactic protein 1.*

a *Independent samples t-test;*

b *Mann–Whitney U-test*.

### The Association Between Cytokines and Size of CNV

**Figure 1:**
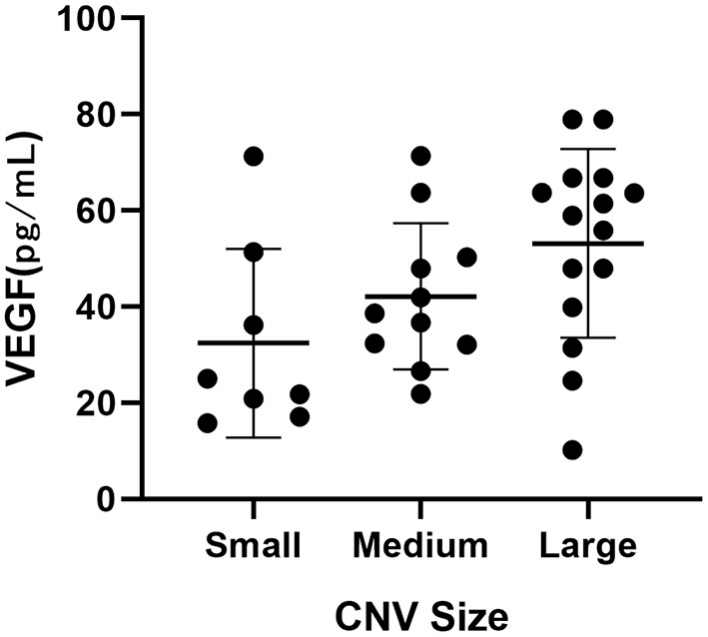
Dot plot showing the correlation between intraocular VEGF levels and CNV sizes on OCTA in AMD patients.

A significant difference was observed among the three CNV size groups (*p* = 0.043, Table 5, Figure 1). The VEGF level increased with the size of CNV.

**Table 5:** Differences in cytokine concentrations (pg/mL) in groups of different vessel size (mm^2^).

	**Small**	**Medium**	**Large**	***P*** **-value**
	** <0.5**	**≥0.5 and <2**	**≥2**	
	**(*n* = 8)**	**(*n* = 11)**	**(*n* = 15)**	
VEGF	32.43 ± 19.62	42.14 ± 15.20	53.14 ± 19.63	0.043^a^
IL-6	7.38 ± 8.56	2.69 ± 1.61	11.07 ± 21.47	0.522^b^
IL-8	10.54 ± 8.13	17.41 ± 17.24	17.41 ± 17.24	0.297^b^
IL-10	0.80 ± 0.38	0.89 ± 0.35	1.15 ± 0.48	0.057^b^
IP-10	306.06 ± 241.69	349.963 ± 257.06	403.57 ± 313.35	0.657^b^
MCP-1	421.11 ± 187.37	414.68 ± 105.51	413.75 ± 158.99	0.665^b^

*VEGF, vascular endothelial growth factor; IL-6, interleukin 6; IL-8, interleukin 8; IL-10, interleukin 10; IP-10, interferon-inducible protein 10; MCP-1, monocyte chemotactic protein 1. P-values calculated by Spearman rank correlation analysis.*

a *One-way ANOVA test;*

b *Kruskal–Wallis test*.

## Discussion

The present study yielded six main findings. First, VEGF levels increased significantly in all CNV diseases except for mCNV. Second, some inflammatory cytokine levels increased significantly in all CNV diseases except for nCSC. Third, the angiogenesis index increased significantly in all CNV diseases, and it may be a more sensitive indicator of the presence of CNV than inflammatory cytokine levels. Fourth, intraocular inflammatory cytokine levels in PCV were quite different from those in AMD. Fifth, the same types of CNV secondary to different causes were not completely similar in cytokine levels. Sixths, VEGF levels may have a positive relationship with CNV size on OCTA.

Our results were consistent with previous studies showing that the ocular concentration of VEGF was elevated in all CNV groups except for mCNV compared with the control group (14, 15). The most plausible explanation for the normal level of VEGF in mCNV is the dilution effect in the anterior chamber and vitreous cavity due to the extended axial length (16).

Choroidal vessel hyperpermeability and congestion were suggested to be the cause of CSC (17), which is a disease in the pachychoroid spectrum. Secondary CNV in CSC is also called pachychoroid neovasculopathy (18), which may also be affected by the dysregulation and hyperpermeability of the choroidal vessels (19). To our knowledge, although previous studies have shown that the VEGF level in CSC eyes without neovascularization was similar to those in normal human eyes (20, 21), no research has investigated the VEGF level in the AH of patients with nCSC. In addition, differences in VEGF levels have been found between acute and chronic CSC eyes, and the VEGF level was positively correlated with typical choroidal abnormalities (12, 22). Our results demonstrated that in the nCSC group, only the level of VEGF increased; other pro-inflammatory cytokines remained unchanged, which supports the current view that the mechanism of neovascularization in pachychoroid disease differs greatly from that in AMD (23). The results also provide a theoretical basis for the treatment outcome reported by another study in which all patients with nCSC exhibited reduced pigment epithelial detachment thickness after 6 months of intravitreal anti-VEGF injection treatment (18). In the nAMD group, the levels of the inflammatory cytokines IL-10 and IP-10 both increased. This could explain why anti-VEGF treatment was equally effective in both diseases but eyes with pachychoroid neovasculopathy required fewer injections for maintenance (24).

In our study, the IP-10 level was elevated in all CNV groups except the nCSC group. IP-10 was capable of inhibiting both retinal and choroidal neovascularization as an inflammation-related chemokine (25). However, the molecular mechanism remains unknown. A previous *in vitro* study suggested that IP-10 inhibits VEGF-mediated m-calpain activation, thereby disrupting any newly formed vessels via chemokine receptor 3 (CXCR3) signaling (26). Fujimura et al. (27) found that CXCR3-mediated angiostasis was independent of the VEGF signaling pathway. The ocular balance between angiogenic and angiostatic factors determines blood vessel formation, which is essential for the progression of neovascularization (28). In line with our results, Liu et al. found that the level of IP-10 was elevated in the AH of AMD patients (20). We speculated that the increase in IP-10 level in naive eyes was a compensatory response to excessively elevated VEGF, but the angiostatic chemokine level was not always high enough to prevent neovascular outgrowth.

MCP-1, as a member of the CC chemokine family, was shown to mediate neovascularization (29). The angiogenesis induced by MCP-1 was as potent as that induced by VEGF *in vivo* angiogenesis assays (28). A positive regulatory feedback loop exists between VEGF and MCP-1 expression by the ocular tissues in mediating angiogenesis (28). VEGF mediates MCP-1-induced angiogenesis, whereas MCP-1 induces VEGF expression via the upregulation of hypoxia-inducible factor 1 alpha gene expression (30, 31). This is a potential mechanism of resistance in anti-VEGF therapy because the anti-VEGF agent only focuses on a single pathogenic mechanism and the high baseline level of MCP-1 could reinduce VEGF expression. For such patients, combined anti-inflammatory therapy or new target agents may be necessary. In addition, we speculate that the co-expression and interrelationship between MCP-1 and IP-10 may have biological effects of angiogenesis regulation, similar to the interrelationship between VEGF and IP-10. We propose the ratio of IP-10 to MCP-1 as a new indicator called the angiogenesis index, which was elevated in all CNV groups in this study. Notably, even if the IP-10 and MCP-1 levels were both elevated in the PCV and mCNV groups, with VEGF at a normal level in mCNV, the angiogenesis index in these groups was still higher than that in the control group. Thus, it may be a more sensitive indicator of the imbalance between the facilitation and inhibition of neovascularization.

It remains controversial whether PCV is an AMD subtype. Recently, a growing consensus is that PCV lies within the pachychoroid disease spectrum, characterized by its hyperpermeable and dilated choroidal vessels. PCV is also a variant of Type 1 (sub-retinal pigment epithelium) neovascularization (19). Tong et al. found that the VEGF levels in PCV eyes were lower than those in exudative AMD eyes (32), but other previous studies observed no significant differences in cytokine levels between patients with AMD and PCV (14, 33, 34). These discrepancies in outcomes may be due to the lack of distinction between Type 1 and Type 2 CNVs. Our study compared PCV with AMD Type 1 CNV and the AMD Type 2 CNV and observed a significant difference in VEGF and IL-10 between the AMD Type 1 and Type 2 CNV groups. The VEGF level in PCV was similar to that in AMD Type 1 CNV, and both were lower than that in AMD Type 2 CNV. In the PCV group, the angiogenesis index and levels of inflammatory cytokines (i.e., IL-8, IL-10, IP-10, and MCP-1) were increased compared with the AMD Type 1 CNV group. This suggests that inflammation plays a role in PCV, which is different from the pathogenic mechanism in AMD.

The variation in the AH concentration of VEGF we found in different types of CNV may explain why different types of CNV showed different responses to anti-VEGF treatment (35). This variation may depend on whether the neovascularization has penetrated the retinal pigment epithelium; the penetration of such a crucial intraocular biological barrier can affect the AH concentration of VEGF and anti-VEGF agents (36).

The same type of CNV, secondary to different causes, is not completely similar in cytokines. In the mCNV Type 2 group, the VEGF level was lower, but the inflammatory cytokines (i.e., IL-6, IL-10, and MCP-1) levels were higher than that in the AMD Type 2 groups, which supported a possible connection between highly myopic eyes and low-grade or subclinical inflammation (37). The IL-6 level increased with the elongation of the eye globe, indicating a connection between inflammation and eye globe elongation (37). Zhao et al. (38) demonstrated that the upregulation of MCP-1 in fibroblasts raised the scleral macrophage density and matrix metallopeptidase 2 levels, which then contributed to axial length elongation and myopia development. However, whether these changes in inflammatory cytokines are a result or a cause of this disease is unclear. Further investigation is required to determine whether anti-inflammatory treatment is effective in patients who are not sensitive to anti-VEGF treatment.

We also observed an association of the intraocular VEGF levels with CNV sizes on OCTA in AMD patients. To our knowledge, no similar results have previously been reported. This is a preliminary result obtained from patients with high-quality images. Further studies are required to validate this finding.

One limitation of this study is the relatively small number of cases. In addition, the location of CNV varied, and the focal changes of cytokines in the intraocular microenvironment may not be well-reflected when they are solely based on AH samples. Finally, only six cytokines were examined in this study, and other cytokines may contribute more strongly to the formation of CNV.

In conclusion, we confirmed the differences in intraocular inflammatory cytokine levels in eyes with different CNV diseases and types. The angiogenesis index may be a more sensitive indicator of angiogenesis than inflammatory cytokine levels. The positive relationship between VEGF and CNV size requires further validation in future studies.

## Data Availability Statement

The original contributions presented in the study are included in the article/supplementary materials, further inquiries can be directed to the corresponding authors.

## Ethics Statement

The studies involving human participants were reviewed and approved by Wenzhou Medical University Affiliated Eye Hospital Ethics Committee. The patients/participants provided their written informed consent to participate in this study.

## Author Contributions

CL, JM, LH, and LS designed the study. CL, SZ, XD, and YC performed the study. XD and YC performed data collection. CL and SZ performed data analysis and interpretation. CL and LS performed manuscript review and revision. All authors read and approved the final manuscript.

## Funding

This work was supported by the Key Project jointly constructed by Zhejiang Province and Ministry (WKJ-ZJ-2037) and Medical and Health Platform Project of Zhejiang Province (2021KY810).

## Conflict of Interest

The authors declare that the research was conducted in the absence of any commercial or financial relationships that could be construed as a potential conflict of interest.

## Publisher's Note

All claims expressed in this article are solely those of the authors and do not necessarily represent those of their affiliated organizations, or those of the publisher, the editors and the reviewers. Any product that may be evaluated in this article, or claim that may be made by its manufacturer, is not guaranteed or endorsed by the publisher.
